# Disseminated Herpes Zoster Virus: A Severe Case Complicated by Radicular Mononeuropathy in a Clinically Immunocompetent Individual

**DOI:** 10.7759/cureus.98834

**Published:** 2025-12-09

**Authors:** Daniel Neri Rosario, Izabela Turcu, Sachin Sapkota, Abirami Rajendiran, Lela Ruck, Jeffrey Sherwood

**Affiliations:** 1 Internal Medicine, Texas Tech University Health Sciences Center El Paso, Paul L. Foster School of Medicine, El Paso, USA; 2 Internal Medicine, Texas Tech University Health Sciences Center El Paso, El Paso, USA; 3 Infectious Disease, Texas Tech University Health Sciences Center El Paso, El Paso, USA

**Keywords:** antiviral agents, case report, chronic kidney disease (ckd), disseminated herpes, motor radiculopathy

## Abstract

Herpes zoster incidence and severity increase with age due to immunosenescence. While typically presenting as a unilateral dermatomal rash, severe forms such as disseminated zoster and segmental zoster paresis can occur, especially in high-risk individuals. Despite the availability of a highly effective recombinant zoster vaccine, vaccination rates remain low in the United States. This case report is significant due to the presentation of rare complications, disseminated herpes zoster and segmental zoster paresis, in an elderly patient with chronic kidney disease (CKD), underscoring the clinical consequences of delayed diagnosis and treatment in this demographic. We report the case of an 80-year-old Hispanic male with CKD who sought care due to a progressively worsening vesicular rash on his left upper extremity. The rash started 10 days earlier and was initially limited to the T1 dermatome. Skin lesions progressed to involve the entirety of the left upper extremity with motor radiculopathy characterized by profound proximal muscle weakness. Varicella zoster virus was confirmed via polymerase chain reaction testing of the skin lesions. He received intravenous acyclovir, wound care, and pain management, resulting in significant improvement of skin lesions but persistent motor weakness at discharge. This case underscores the potential severity of herpes zoster in high-risk populations such as elderly patients with CKD. It highlights the critical importance of prompt diagnosis, early antiviral intervention (intravenous for severe cases), and monitoring for neurological complications. The suboptimal vaccination rates emphasize the need for improved zoster vaccine uptake, especially in vulnerable individuals, to prevent severe disease and associated morbidity.

## Introduction

Herpes zoster (HZ) reactivation can lead to severe complications, including disseminated disease and segmental zoster paresis [1]. While these severe manifestations are well-recognized in patients with classical immunosuppression (e.g., HIV, malignancy, or iatrogenic suppression), their risk in the much more extensive population of patients with functional immunosuppression is less clearly defined.

Chronic kidney disease (CKD) is an independent risk factor for HZ. A large-scale, retrospective cohort study demonstrated that patients with CKD have a 1.4-fold higher incidence of HZ than non-CKD controls (8.76 vs. 6.27 per 1,000 person-years), with an adjusted hazard ratio of 1.38 (95% confidence interval = 1.25-1.53) [2]. While advanced age naturally leads to immunosenescence, the uremic environment of CKD further impairs cellular immunity, creating a state of functional immunosuppression even in patients without a history of transplant or iatrogenic immunosuppression. Consequently, these patients are vulnerable to severe, atypical viral reactivations that extend beyond the classic dermatomal distribution [3,4].

Disseminated HZ, defined as involvement of more than two adjacent dermatomes or skin lesions crossing the midline, is a serious complication typically associated with profound immunocompromise. Even rarer is segmental zoster paresis, a motor radiculopathy resulting in focal limb weakness, which occurs in approximately 0.5% to 5% of HZ cases [5].

This case reports an 80-year-old male with CKD, but without classical pharmacologic immunosuppression, who developed disseminated HZ complicated by severe segmental paresis. While disseminated herpes zoster with motor complications is well recognized in overtly immunocompromised populations (human immunodeficiency virus (HIV), malignancy, iatrogenic immunosuppression), recognition of these severe manifestations in hosts with compromised immunity due to other conditions, particularly elderly patients with CKD, remains clinically important, as age-related immune decline combined with CKD-related immune dysfunction can lead to severe manifestations.

## Case presentation

An 80-year-old Hispanic male with a medical history of CKD Stage IIIb (estimated glomerular filtration rate (eGFR) 24 mL/minute/1.73m²), coronary artery disease, atrial fibrillation on apixaban, and hypertension developed a rash on his left arm associated with numbness and weakness. Family history was only notable for hypertension in relatives. There was no documented history of depression, anxiety, or other psychiatric illness. He had not completed the vaccination series for HZ as an adult.

He first developed a vesicular rash involving the upper part of his left extremity 10 days before hospitalization, which was initially limited to the T1 dermatome. Based on these characteristic findings, the patient was clinically diagnosed with shingles by his primary care physician and prescribed valacyclovir and gabapentin. However, he did not start these medications due to unclear circumstances. Over the 10-day period between the onset of his symptoms and his seeking care in our emergency department (ED), the skin lesions progressed down the upper extremity and became increasingly painful. Some of the vesicles coalesced, forming large blisters. The patient reported severe pain and progressive weakness in the affected limb during this period.

**Figure 1:**
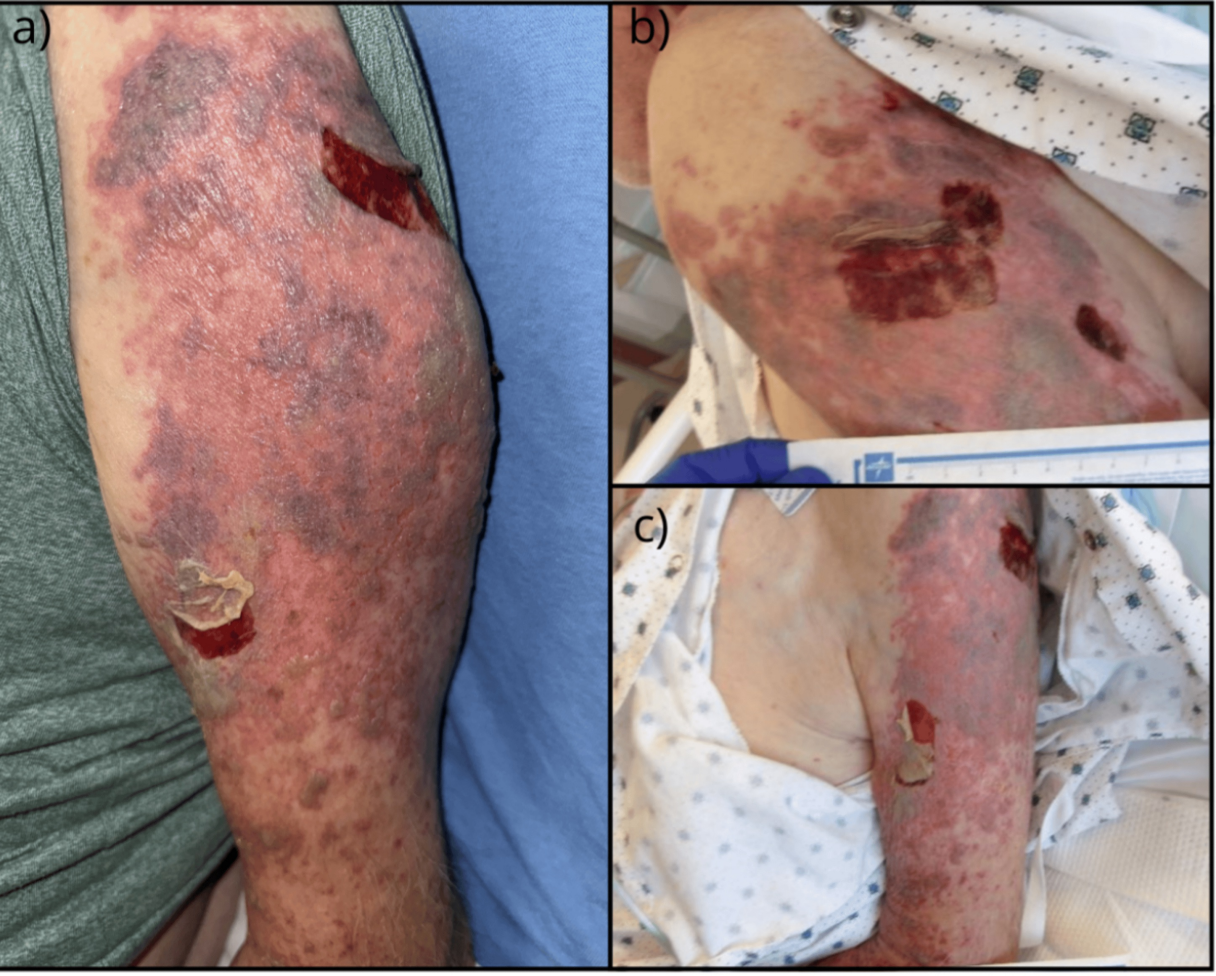
Clinical presentation of the left upper extremity with disseminated herpes zoster. (a) Presentation (day zero): View of the left arm demonstrating the acute hemorrhagic phase. Note the large (2 × 5 cm) tension bullae and coalesced necrotic ulcers localized to the C5-C7 dermatomes. The surrounding skin shows severe erythema, non-pitting edema, and ecchymosis. (b, c) Response to therapy (day two): Clinical appearance 48 hours after the initiation of intravenous acyclovir. The images show a marked reduction in erythema and edema. The hemorrhagic bullae have begun to flatten, and continued presence of the ulcerative lesions.

Upon arrival at our ED on hospital day one, the patient was hemodynamically stable and saturating well on room air. On physical examination, he was noted to have multiple tense, hemorrhagic bullae with discrete coalescing ulcers involving multiple dermatomes of his left upper extremity (T1, C3, and C6). The largest measured approximately 2 × 5 cm. These lesions were surrounded by extensive ecchymoses in varying stages (purple and yellow discoloration), significant erythema, and diffuse, non-pitting edema throughout the affected limb. The patient also had several small, approximately 5-7 mm vesicular lesions (less than 1 cm) on his upper back. This clinical presentation, consistent with disseminated zoster, is shown in Figure 1a.

Due to the extent of skin lesions and significant pain involving his left upper extremity, neurologic examination was limited. Motor strength was severely impaired (1/5 on the Medical Research Council scale) throughout the limb, and deep tendon reflexes at the biceps, triceps, and brachioradialis were absent. While sensory examination was also restricted, sensation to light touch and pinprick appeared intact. The patient reported significant tingling and burning pain in the affected limb consistent with neuralgia. The contralateral right upper extremity demonstrated normal neurologic function, including full strength (5/5), intact reflexes, and normal sensation, with no reported pain or paresthesia. Laboratory findings revealed an elevated creatinine of 2.6 mg/dL and a blood urea nitrogen of 62 mg/dL (Table 1). This acute-on-chronic renal insufficiency (baseline eGFR ~24 mL/minute) underscored the patient’s state of functional immunosuppression and necessitated precise renal dosing of intravenous acyclovir to prevent neurotoxicity. The complete blood count demonstrated mild leukocytosis, with a white blood cell count of 10.02 × 10³/μL, predominantly neutrophilic (absolute neutrophil count of 6.40 × 10³/μL), consistent with the acute inflammatory response. Liver function tests revealed mild elevations in aspartate aminotransferase (AST) and alanine aminotransferase (ALT) to 42 U/L. In the context of the vesicular eruption, this transaminitis likely reflected visceral dissemination of the varicella-zoster virus.

**Table 1:** Patient’s initial laboratory findings. Abnormal laboratory findings supporting a diagnosis of chronic kidney disease and systemic inflammation.

Parameter	Patient’s value	Normal range	Units
Creatinine	2.6	0.7–1.3	mg/dL
Blood urea nitrogen	62	6–20	mg/dL
Estimated glomerular filtration rate	24	>60	mL/minute/1.73m²
White blood cell count	10.02	4.5–11.0	10³/µL
Neutrophils (%)	63.8	40–60	%
Aspartate aminotransferase	42	10–40	U/L
Alanine aminotransferase	42	7–56	U/L

The patient received supportive care, including hydration and pain management. Given the concern for disseminated HZ, he was admitted to the medical floor. Wound care and infectious disease services were consulted. Given the high clinical suspicion for disseminated HZ, intravenous acyclovir was initiated empirically (10 mg/kg every eight hours with dose adjusted for renal function) along with intravenous ceftriaxone for empiric skin and soft tissue coverage. The patient did not have any side effects or withholding. Due to the presence of hemorrhagic lesions, the patient’s outpatient anticoagulation with apixaban (Eliquis) was temporarily withheld.

The patient reported the pain associated with the rash as a 10 out of 10 on the pain scale. A multimodal pain management regimen, consisting of scheduled gabapentin and as-needed acetaminophen and tramadol, was initiated. On the second day of his hospitalization, the patient reported significant improvement in left arm pain accompanied by a marked reduction in edema and erythema (Figures 1b, 1c). Despite this improvement, physical examination revealed persistent proximal muscle weakness, and the patient continued to report paresthesia in the affected extremity. CT of the left upper extremity was ordered to investigate the severe proximal weakness, which showed no significant findings. Further evaluation included negative HIV screening and normal IgG levels. Polymerase chain reaction (PCR) testing of skin lesions subsequently returned positive for VZV, confirming the diagnosis of disseminated zoster and effectively ruling out herpes simplex virus. Liver function tests revealed mild elevations in AST and ALT to 42 U/L. In the context of the vesicular eruption, this transaminitis likely reflected visceral dissemination of the VZV.

**Figure 2:**
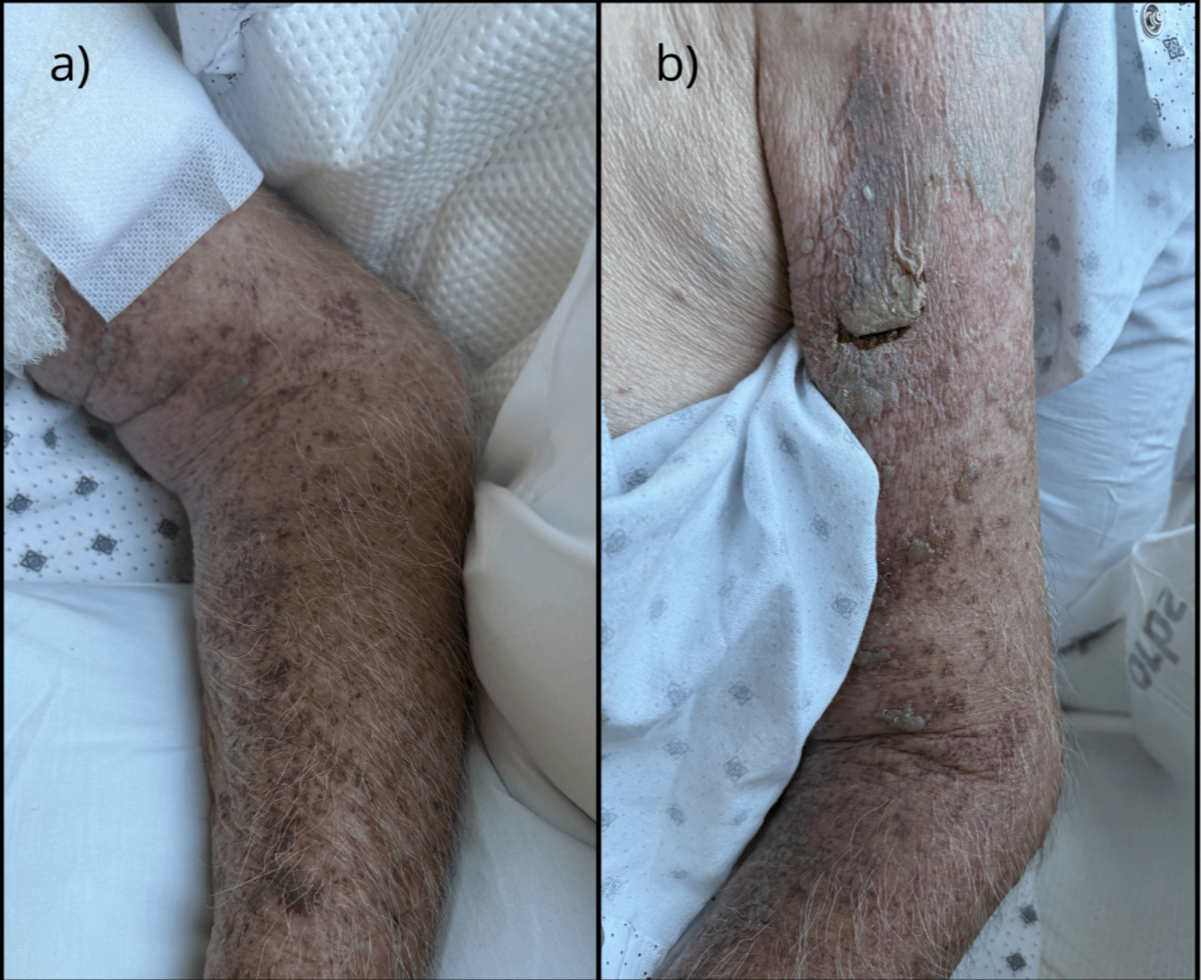
Clinical improvement of disseminated herpes zoster in the left upper extremity after four days of intravenous acyclovir treatment. (a, b) Discharge (day four): Clinical appearance before discharge. The hemorrhagic bullae have resolved into crusted plaques. The erythema, edema, and ecchymosis have significantly reduced, and no new vesicle formation is noted.

By the fourth day of hospitalization, while he acknowledged improvement in his left arm pain with multimodal pain management, the persistent weakness was concerning him. Skin lesions also demonstrated substantial improvement by discharge (Figures 2a, 2b). Physical and occupational therapy were initiated during his hospital stay, with recommendations for continued therapy after discharge to address ongoing weakness. The patient was released with prescriptions for gabapentin and venlafaxine for ongoing neuropathic pain. He was also provided an additional 10-day course of oral acyclovir, with the dosage appropriately adjusted for renal function.

## Discussion

By definition, active involvement of more than one dermatome represents disseminated VZV. In cases where the clinical diagnosis of VZV is uncertain or atypical, PCR testing of skin lesions can provide a sensitive and specific diagnostic tool [5]. Antiviral treatment such as acyclovir or valacyclovir has significant efficacy in mitigating the course of HZ, especially if initiated early during the course of the infection [6].

The route of administration of these agents, whether oral or intravenous, depends on the severity of the disease and the patient’s clinical status. In patients with disseminated HZ, intravenous antiviral therapy is the appropriate initial route [6]. The Kidney Disease: Improving Global Outcomes guideline recommends intravenous acyclovir for disseminated or invasive HZ in patients with severe CKD. Once the patient demonstrates a clinical response, the treatment can be transitioned to an appropriate oral antiviral equivalent such as acyclovir, valacyclovir, or famciclovir [7].

CKD has been identified as an independent risk factor for the development of HZ. A matched-for-age and sex cohort study indicated that CKD was associated with a 1.60-fold increased risk of HZ [8, 9]. This increased susceptibility is multifactorial, including cellular immune dysfunction related to CKD, malnutrition, volume overload, and the accumulation of uremic toxins [10]. The prevalence of CKD represents a substantial public health concern in the United States, affecting almost 14% of adults in the United States [11]. As CKD progresses, patients are at an increased risk of infections, which are the second most common cause of morbidity and mortality in this population [12].

While postherpetic neuralgia is a prevalent neurological complication of HZ, motor involvement occurs in only 0.5% to 5% of cases. Segmental zoster paresis and paralysis syndromes are well-documented and potentially debilitating manifestations of the disease that have been described mostly at the case report level [5,13,14]. Recovery can be prolonged. The mainstay of treatment for HZ-related motor radiculopathy includes timely administration of antiviral medication to limit viral replication and neuronal damage, effective pain management strategies, and a comprehensive physical and occupational therapy program to facilitate recovery of motor function [5]. Brachial plexus involvement, specifically in HZ, is believed to result from the distal extension of the inflammatory process along the sensory ganglion and into the adjacent motor nerve roots [13].

Some studies have explored the potential benefit of adjunctive corticosteroids, in combination with antivirals, in reducing acute pain and accelerating the healing of motor deficits. However, the evidence remains contentious, and such an approach should be taken with caution, especially in patients with immunosuppression. Recent Cochrane and meta-analytic reviews note that sample sizes have been small, with moderate to high risk of bias, and significant heterogeneity in patient populations and corticosteroid dosing regimens [15]. Particular caution is warranted in elderly patients and underlying immune dysfunction, who are at an increased risk for serious adverse events from corticosteroids.

## Conclusions

This case illustrates the concept of functional immunosuppression in elderly patients with CKD, illustrating that severe complications such as disseminated zoster and segmental paresis can occur even in the absence of classical immunosuppressive therapy. The progression of disease observed here highlights the critical importance of early antiviral initiation and neurologic monitoring in elderly CKD patients who may not be traditionally considered immunocompromised but remain at high risk for severe zoster complications. While the diagnosis of segmental zoster paresis was established clinically, a limitation of this report is the absence of confirmatory electrodiagnostic testing (electromyography/nerve conduction study) and long-term follow-up data regarding motor recovery. Nevertheless, the temporal association between the dermatomal rash and the onset of profound focal weakness serves as a vital alert for clinicians. Recognition of this rare motor complication is essential to prevent diagnostic delays. Finally, this case underscores the urgency of improving zoster vaccination rates in the CKD population, as prevention remains the most effective strategy against the morbidity associated with disseminated disease and motor radiculopathy.
